# Molecular Evidence that *Lysiphlebia japonica* Regulates the Development and Physiological Metabolism of *Aphis gossypii*

**DOI:** 10.3390/ijms21134610

**Published:** 2020-06-29

**Authors:** Xueke Gao, Hui Xue, Junyu Luo, Jichao Ji, Lijuan Zhang, Lin Niu, Xiangzhen Zhu, Li Wang, Shuai Zhang, Jinjie Cui

**Affiliations:** 1 Institute of Cotton Research, Chinese Academy of Agricultural Sciences, Anyang 455000, China; 15036138389@163.com (X.G.); luojunyu1818@126.com (J.L.); hnnydxjc@163.com (J.J.); zhlj042@126.com (L.Z.); nl19882006@126.com (L.N.); zhuxiangzhen318@163.com (X.Z.); wangli08zb@126.com (L.W.); 2 Zhengzhou Reseach Base, State Key Laboratory of Cotton Biology, Zhengzhou University, Zhengzhou 4550001, China; 3 College of Plant Science and Technology, Huazhong Agricultural University, Wuhan 430070, China; xuehuihzau@163.com

**Keywords:** proteome, transcriptome, parasitism, immunization, lipid

## Abstract

*Lysiphlebia japonica* Ashmead (Hymenoptera, Braconidae) is an endophagous parasitoid and *Aphis gossypii* Glover (Hemiptera, Aphididae) is a major pest in cotton. The relationship between insect host-parasitoids and their hosts involves complex physiological, biochemical and genetic interactions. This study examines changes in the development and physiological metabolism of *A. gossypii* regulated by *L. japonica*. Our results demonstrated that both the body length and width increased compared to non-parasitized aphids. We detected significantly increases in the developmental period as well as severe reproductive castration following parasitization by *L. japonica*. We then used proteomics to characterize these biological changes, and when combined with transcriptomes, this analysis demonstrated that the differential expression of mRNA (up or downregulation) captured a maximum of 48.7% of the variations of protein expression. We assigned these proteins to functional categories that included immunity, energy metabolism and transport, lipid metabolism, and reproduction. We then verified the contents of glycogen and 6-phosphate glucose, which demonstrated that these important energy sources were significantly altered following parasitization. These results uncover the effects on *A. gossypii* following parasitization by *L. japonica*, additional insight into the mechanisms behind insect-insect parasitism, and a better understanding of host-parasite interactions.

## 1. Introduction

Parasitoids naturally parasitize insects and can serve as important agents of biological control in agricultural ecosystems. Endoparasitoids oviposit into the hemocoel of their insect hosts. When the eggs hatch, the parasitoid larvae consume the host tissues and the hemolymph [1,2]. Some parasitoid eggs (or larvae) have unique surface features that passively evade hemocyte encapsulation in the host [3]. Other parasitoids can actively interfere with host immune defenses using venom, polydnaviruses (PDVs), virus-like particles (VLPs), teratocytes, ovarian proteins, or parasitoid larval secretions [4,5,6,7,8]. Ectoparasitoid venom is involved in host paralysis (either short or long-term) to ensure they can feed the ectoparasitic larva outside the host. This is accomplished by interfering with the host immune system or host development, or synergizing the effects of other maternal factors introduced into the host [9].

The relationship between the host and the parasitoid is complex. Parasitoid venom can disrupt host immune responses [6,10], but also stimulate host defense responses, particularly hemocytic encapsulation and the generation of reactive oxygen species [11]. Insects have several defense mechanisms, including encapsulation and production of antimicrobial substances [12,13,14]. Different parasitoid species lack the ability to synthesize lipids [15,16,17,18,19,20], but lipids and energy reserves play key roles in their survival and reproduction. Significant changes in the host lipids and energy metabolism have been observed [21,22], while the major metabolic pathways associated with sugar and lipid metabolism are typically highly conserved [23,24]. Another important host change caused by parasitoids is host castration, which interferes with host reproductive development and increases the available energy resources available from the hosts. This induces parasitoid larval growth as host nutritional increase [25,26]. Endoparasitoid wasps such as *Aphidius ervi* counter host defenses and create an environment suitable for wasp development using both maternal and embryonic factors [9]. γ-glutamyl transpeptidase is a venom protein used by A. ervi larvae to induce apoptosis in the host ovarioles [27].

Parasitic virulence factors associated with venom and salivary glands which include bioactive peptides, proteins, and enzymes. Considerable progress has been reported in understanding the roles of these molecules [5,7,28]. Additionally, many host genes are altered following parasitization [29,30]. Parasitoids use a variety of methods to commandeer their insect hosts and create an environment that will support and promote their own development [9]. Changes in the hemolymph profile of lipids, proteins, and carbohydrates have been observed in host insects altered by PDVs, VLPs, teratocytes, and venoms [27,31,32]. However, there are few resources detailing hosts that have been manipulated by parasitoids.

In this study, we examined the development of *A. gossypii* regulated by *L. japonica* at various life stages. In addition to providing comprehensive biological changes, we used mass-spectrometry-based proteomics combined with RNA sequencing to analyze the effects of the parasitoid *L. japonica* on the protein and gene expression of *A. gossypii*. The correlation between proteome and transcriptome data was analyzed to comprehensively understand this regulation. Additionally, we analyzed the contents of glycogen and 6-phosphate glucose, which were key substances in glycolysis. These datasets will be useful in future studies researching changes in protein and gene expression in parasitized *A. gossypii*.

## 2. Results

### 2.1. Changes in Development on Aphids Parasitized by L. japonica

The effects of *L. japonica* on the body length and width during different stages of parasitized cotton aphids are shown in Table 1. The results demonstrate that the body length and width of aphids increased as the duration of parasitism increased, reaching a significant difference after 3 days of parasitism (*p* < 0.05). Compared with the non-unparasitized aphids, the body length and width increased by an average of 0.08–0.19 mm and 0.07–0.14 mm, respectively, after parasitization for 1, 2, 3, 4, and 5 d. The effects of *L. japonica* on the developmental periods of parasitized cotton aphids are shown in Table 2. Compared with the non-parasitized aphids, the time from molting until the next stage in parasitized aphids was increased (with average extensions of 10.10 h), and significant changs were observed after 1 day of parasitization. Additionally, parasitism decreased the reproductive capacity of cotton aphids (Table 3): following parasitization, the average number of offspring numbers of freshly hatched nymphs, 1st, 2nd, 3rd, and 4th aphids were 0.67, 1.68, 4.52, 7.36, and 9.60, respectively. While the average number of offspring of non-parasitizated aphids was 32.9.

### 2.2. Primary Data Analysis and Protein Identification

A total of 332, 968 spectra were obtained in our study (Figure S1A), which includes 38, 015 unique spectra, 17, 130 identified peptides (16259 unique peptides), and 4, 568 identified proteins. The protein mass distribution above 10 kDa is normally distributed (Figure S1B), where 10–50 kDa accounts for 65%, 50–100 kDa for 28%, and over 100 kDa for 7%. The proteins of a single peptide, 2–5 peptides, 6–10 peptides and 11 peptides were composed of 1, 911, 1, 810, 570 and 277 peptides, respectively (Figure S1C). Additionally, the protein sequence coverage was assessed (Figure S1D), where the protein sequence coverage of 40–100, 30–40, 20–30, 10–20, and less than 10% was 3.57, 1.56, 3.36, 8.04, and 83.4% coverage, respectively.

### 2.3. Functional Categories of Proteins Associated with mRNA

GO analysis was performed according to the Gene Ontology Annotation in order to classify the proteins based on their biological processes (3, 132, 41.1%), cellular localization (3, 033, 39.8%), and molecular function (1453, 19.1%). A total of 19 molecular function groups were delineated (Figure S2A). The metabolism processes (698 proteins, 22.2%), cellular processes (680 proteins, 21.7%), single-organism processes (293 proteins, 9.3%), and the response to stimulus groups (266 proteins, 8.5%) were the most abundant. The developmental process, cellular component organization or biogenesis, and localization groups included almost 5% of all the proteins identified. The proteins identified using mRNA analysis were classified based on their cellular localization (Figure S2B). A total of 1, 540 proteins (50.7%) were localized in the cytoplasm and associated parts, while 554 (18.2%), 208 (6.8%), and 234 (7.7%) proteins were localized in the organelle, organelle parts, and macromolecular complexes, respectively. In particular, 257 proteins (8.5%) proteins were localized in the membrane. Membrane-associated proteins are important to the functioning of the organism, including cellular signaling and recognition. Classification based on molecular function demonstrated that the majority of the identified proteins were involved in catalytic activity and binding activity (Figure S2C), while 759 (52.2%) and 555 (38.2%) of the proteins were localized, respectively.

There were 3, 191 proteins were assigned to COG classifications, with the groups categorized as follows: general function prediction only (17.4%), post-translational modification, protein turnover, and chaperones (11.1%), translation, ribosomal structure, and biogenesis (10.3%) energy production and conversion (6.5%), lipid transport and metabolism (5.9%), amino acid transport and metabolism (5.8%), and carbohydrate transport and metabolism (5.0%) (Figure S2D). We assigned 118 KEGG pathways to identify the biological pathways active in parasitized and non-parasitized aphids, and the highest group was metabolic pathways (14.2%). Pathways with the 3 primary degrees of representation were carbohydrate and energy metabolism (239 members), lipid metabolism (114 members), and the digestive system (131 members).

### 2.4. Correlation Analysis of mRNA and Protein Levels

For proteins prepared from parasitized and non-parasitized aphids, we defined proteins with a ratio of >1.2-fold as up-regulated, and proteins with a ratio of <0.8 as down-regulated (*p* < 0.05). Genes with a ratio of >1.5-fold were considered up-regulated, and genes with a ratio of <0.5 were considereddown-regulated (*p* < 0.05).

**Figure 1:**
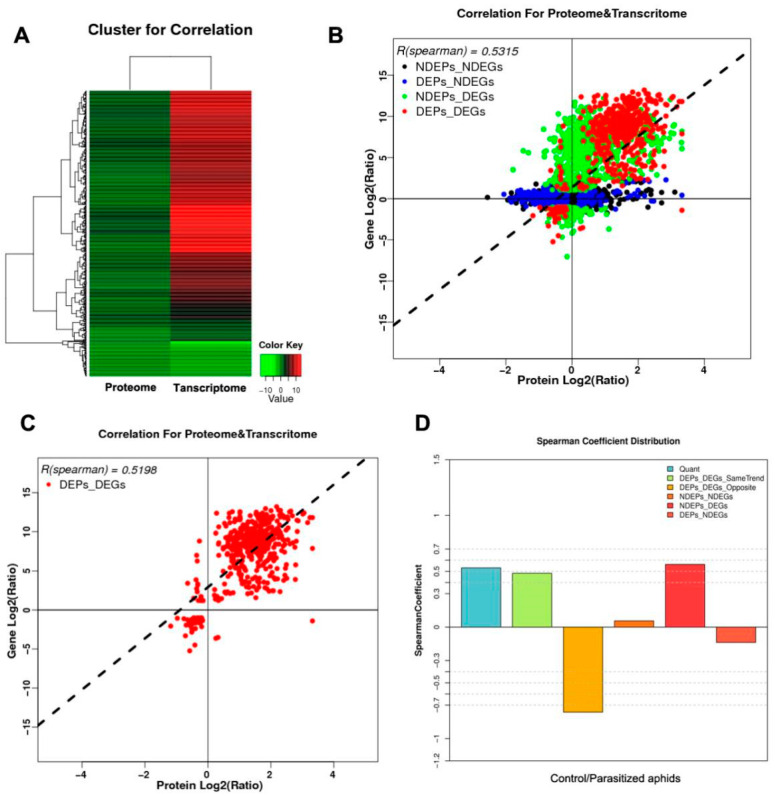
Correlations of mRNA and protein abundance between and within parasitized aphids and non-parasitized aphids. (**A**) Different expression proteins and gene patterns in response to parasitization in aphids were classified using hierarchical clustering. Each row in the figure represents a protein/gene, with different colors representing different fold magnitudes. Red represents upregulation and green represents downregulation; (**B**) Quantitative protein and gene expression association diagram showing the two samples of the proteins and genes and a graph of the quantitative analysis of the correlation. The abscissa represents the protein expression and the ordinate represents the expression level of the gene. Black spots show no significant difference between the mRNA and the protein, blue dots show no significant difference in mRNA and significant differences in the protein, green points show significant differences in the mRNA and no significant differences in the protein, red spots show that the mRNA and the protein were significantly different; (**C**) Significant differences in protein and in gene expression are shown in the two samples in the gene-mapping correlation analysis, where the abscissa represents the protein and the ordinate represents the expression level of the gene; (**D**) The distribution of Spearman’s correlation coefficient of the mRNA and protein. The correlation coefficient for quantitative and quantitative association results is classified into five categories. The abscissa represents different alignment groups, and the ordinate represents the correlation coefficient. Each group has six types of correlation coefficients, which were the result of the correlation between all quantitative protein and mRNA.

Different expression proteins and gene expression patterns responding to aphid parasitism were classified using hierarchical clustering (Figure 1A). We identified a total of 1056 signature genes with significant changes (*p* < 0.01) at either the protein and/or the mRNA level. The correlation coefficient between the mRNA and the proteins was 0.52 (Figure 1B) for the signature genes and 0.53 for all the genes we assessed. The 539 signature genes (51.04%) exhibited changes for mRNAs and their cognate proteins in the same direction (Figure 1C); 518 changed significantly at both the mRNA and protein levels and were considered correlated genes (red). In contrast, 1, 005 genes displayed significant changes at the mRNA level but not the protein level (green), while 518 genes displayed significant changes at the protein level but not the mRNA level (blue). Twenty-one genes displayed opposite expression patterns of mRNA and protein (brown). The distribution of Spearman’s correlation coefficient for both the mRNA and the proteins are shown in Figure 1D. GO enrichment correlation and KEGG enrichment correlation are shown in Figure S3.

### 2.5. Differentially Expressed Proteins and Genes Associated with Immunization of A. gossypii

**Figure 2:**
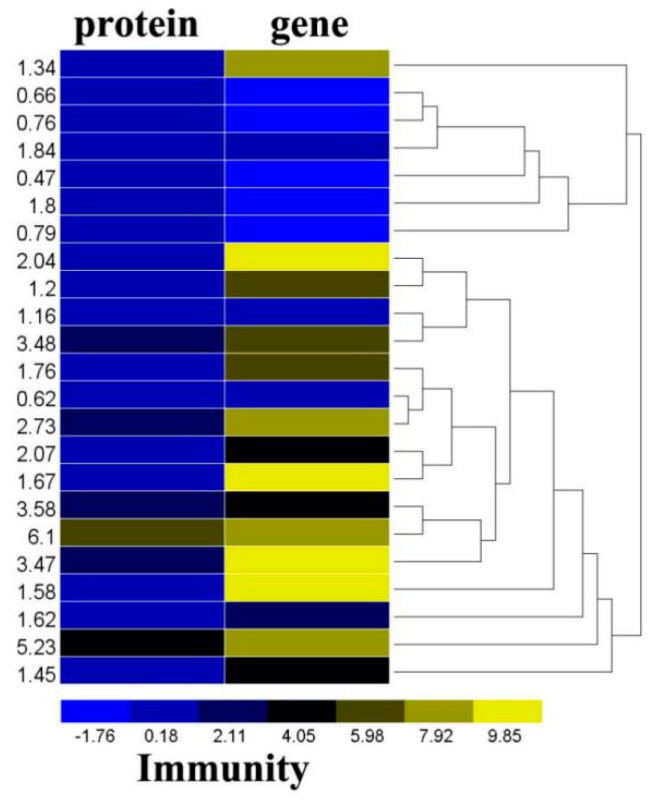
Differentially expressed proteins and genes associated with immunization of *A. gossypii.* Fold change of the differentially expressed proteins and the log2 fold change of the differentially expressed gene was clustered using the R software.

Analysis of the proteomic and transcriptomics profiles demonstrated that the expression of two major immune regulatory proteins (serine protease (protein = 1.62, gene = 3.09) and its inhibitor, serpinB (protein = 1.16, gene = 1.68) increased following parasitism. Two SOD (superoxide dismutase, Cu/Zn superoxide dismutase (protein = 1.67, gene = 9.90), Fe/Mn superoxide dismutase (protein = 2.63, gene = 9.71)) and the related genes were up-regulated in parasitized aphids. Two lectins (lectin, mannose-binding 1/2, protein = 1.58/1.84, gene = 10.65/0.33), two galectins (Galectin-4 (protein = 1.45, gene = 4.51) and Galectin-9 (protein =6.1, gene = 8.47) were significantly up-regulated in parasitized aphids. Additionally, Calreticulin (protein = 3.47, gene = 11.7) were significantly increased at both the protein and transcript levels (Figure 2, Table S1 ).

### 2.6. Differentially Expressed Proteins and Genes Associated with A. gossypi Energy Metabolism

#### 2.6.1. Amino Acid and Protein Transport and Metabolism

**Figure 3:**
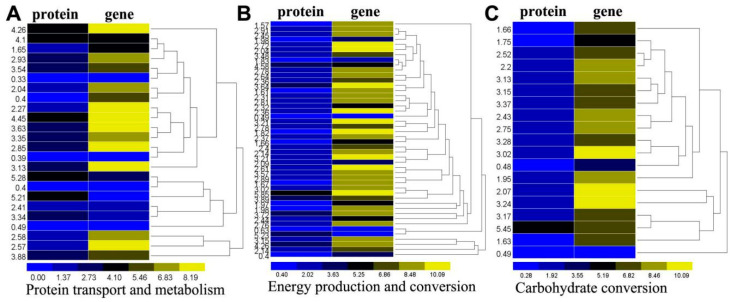
Differentially expressed proteins and genes associated with energy transport of *A. gossypii.* Fold change of the differentially expressed proteins and the log2 fold change of differentially expressed genes were clustered using the R software. (**A**) Differentially expressed proteins and genes associated with amino acid and protein transport and metabolism; (**B**) Differentially expressed proteins and genes associated with energy production and conversion; (**C**) Differentially expressed proteins and genes associated with carbohydrate production and conversion.

We observed that the majority of both amino acid and protein transporters were up-regulated in parasitized aphids, which suggests athey are involved in active transportation processes (Table S1). This indicates that the biosynthesis of proteins was increased in parasitized aphids. Translation initiation factor (TIF) 3 and TIF4 alpha subunit and genes were both up-regulated in parasitized aphids. Glutamine synthase isoform 1 (GS1) and glutamate dehydrogenase (GDH) revealed that glutamine metabolism was up-regulated and ubiquitin cascade-related proteins were differentially regulated. In the case of the ubiquitin cascade, we observed that the ubiquitin-activating enzyme (E1) and the ubiquitin conjugating enzyme (E2) were up-regulated, while and ubiquitin ligase (E3) was down-regulated at the protein level but up-regulated at the transcript level (Figure 3A). This suggests the presence of a rate-limiting mechanism or controlled proteolysis through ubiquitin-mediated protein degradation during parasitization. We also observed that LSD1 (lysine-specific histone demethylase 1, protein = 0.40, gene = 5.57), enolase-phosphatase E1 (protein = 5.23, gene = 1.00) and one oligopeptide transporter (protein = 5.21, gene = 0.51) displayed a reverse expression pattern at the protein and mRNA levels, which demonstrated that protein abundance depends not only on the transcript level, but also on post-translational modification [33].

#### 2.6.2. Energy and Carbohydrate Production and Conversion

After analyzing the proteomic and transcriptome profiles, we determined that different expressions of proteins and genes involved in energy production were up-regulated in the parasitized aphids, with the exception of alpha-glucosidase, citrate synthase and 6-phosphogluconate dehydrogenase, which are involved in glycolysis system. These proteins are mainly comprised of succinate-semialdehyde dehydrogenase that are involved in the acid cycle (TCA) system, and hexokinase, glyceraldehyde 3-phosphate dehydrogenase, mannose-6-phosphate isomerase, triosephosphate isomerase, 6-phosphofructo-2-kinase, pyruvate kinase and glucose-6-phosphate isomerase that qre involved in the glycolysis system. One sugar-activated enzyme (UDP-N-acetylglucosamine pyrophosphorylase, protein = 1.61, gene = 9.18) was identified at both the high protein and gene expression levels in parasitized aphids (Figure 3B,C).

#### 2.6.3. Lipid Transport and Metabolism

**Figure 4:**
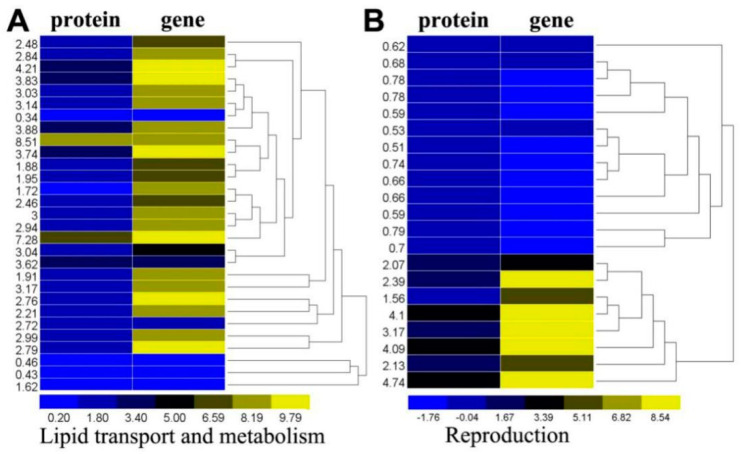
Differentially expressed proteins and genes in *A. gossypii.* Fold change of the differentially expressed proteins and the log2 fold change of differentially expressed genes were clustered using the R software. (**A**) Differentially expressed proteins and genes associated with lipid transport and metabolism; (**B**) Differentially expressed proteins and genes associated with the reproduction of *A. gossypii.*

The expression of several proteins related to lipid transport and metabolism in parasitized aphids was altered (Figure 4A, Table S1). The majority of the significantly changed proteins and genes were involved in phospholipid metabolism and fatty acid metabolism, including one enzyme (sterol carrier protein 2) related to sterol transfer, phospholipid-transporting ATPase, acetyl-CoA acyltransferase, Lysophosphatidic acidacyltransferase, 1-alkyl-2-acetylglycerophosphocholine esterase involved in phospholipid metabolism and transport and long-chain acyl-CoA synthetase, Acyl-CoA dehydrogenases, 3-hydroxyacyl-CoA dehydrogenase, enoyl-CoA hydratase, and stearoyl-CoA desaturase (delta-9 desaturase) involved in fatty acid metabolism, were all significantly increased at the protein and gene levels.

### 2.7. Differentially Expressed Proteins and Genes Associated with Reproduction of A. gossypii

Several proteins and genes associated with development and reproduction were altered in parasitized aphids (Figure 4B, Table S1). Two proteins, the RNA-binding protein (Musashi, protein = 0.78, gene = −1.06) and the serine/threonine-protein kinase (Chk1, protein = 0.59, gene = −1.16) regulate the cell cycle and had significantly reduced protein levels and transcript levels. One juvenile-hormone esterase (protein = 4.10, gene = 9.91) was significantly increased at both the protein and gene levels. An insignificant decrease in the vitellogenic carboxypeptidase-like protein (protein = 0.93, gene = 0.36) protein level was observed. Additionally, three fatty acid-binding proteins (FABP) were significantly increased at both the protein and mRNA levels. These proteins could affect normal development processes and eliminate the reproductive capacity of aphids following parasitization. The γ-glutamyl transpeptidase, which is a parasitoid venom protein, displayed no change at either the protein or transcript level.

### 2.8. The Contents of Glycogen and G6P in Parasitized and Non-Parasitized Aphids

**Figure 5:**
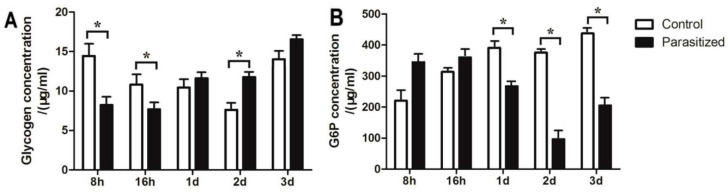
(**A**) Glycogen and (**B**) G6P content of *A. gossypii* after been parasitization by *L. japonica* (* *p* < 0.05).

Our results demonstrated that the glycogen contents of in the parasitized cotton aphids was significantly lower than in the non-parasitized cotton aphids following 8 and 16 h of parasitization. After 1 and 3 days of parasitization, the glycogen content of cotton aphids was higher in control (Figure 5). After 8 h and 16 h of parasitization, the G6P was higher than that in non-parasitized cotton aphids. the 6-phosphoglucose contents was significantly lower in the control aphids after 1, 2, and 3 days of parasitization (Figure 5).

## 3. Discussion

Omics-based technologies, such as transcriptomics and proteomics, have been used in many studies [34]. Analyzing the correlation between the proteome and the transcriptome is necessary to assess multi-level regulation of gene expression [35,36]. In this study, we analyzed the influence of the parasitoids on the development and physiological metabolism of aphids, which provided additional insight into how wasps regulate intracellular host metabolism to meet the needs of the parasitoid growth cycle. Significant changes were associated with several functional classes including immunity, stress and detoxification, energy metabolism and transport, development and reproduction, and lipid metabolism and transport.

The immune system of D. melanogaster is an appropriate model to help understand natural immunity in mammals [37,38]. In contrast, little is known about the signaling pathways involved in anti-parasitic responses and the molecules involved in parasite recognition. In this study, two major immune regulatory proteins (serine protease and its inhibitor serpin) were increased in parasitized aphids, which have been reported in several insects [29,39]. GSTs and SODs play central roles in the detoxification of endogenous and xenobiotic compounds and combat reactive oxygen species generated by oxidative stress [40] and were up-regulated following parasitization. Both lectins and alectins, which act by binding to polysaccharide chains on the surface of pathogens, were up-regulated in parasitized *A. gossypii* [41]. These changes demonstated host response to parasitization [42,43]. Additionally, Calreticulin (protein = 3.47, gene = 11.7), which can inhibit hemocyte spreading behavior, was significantly increased at both the protein and transcript levels. This prevented the encapsulation of the developing parasitoid [44].

The relationship between parasitoids and their hosts is complex and involves not only stress adaptation and immune responses, but also additional energy production in the host at both the mRNA and protein levels [9,29]. Translation initiation factor (TIF) 3 and TIF4 were up-regulated. These proteins participate in the initiation phase of eukaryotic translation during assembly of ribosomes [45]. With regards to energy and carbohydrate production and conversion in *A. gossypii*, some proteins involved in the TCA cycle and glycolysis were up-regulated at the protein and mRNA levels following parasitization. Increased glycolysis activity has been observed in other parasitized aphid species [21]. The low protein levels of the pace-making enzyme citrate synthase and the up-regulated gene were consistent with research demonstrating that citrates in parasitized *A. gossypii* are derived from amino acids [21,46]. Glycogen is the main carbohydrate energy storage substance used by insects. In most cases, parasitic wasps cause an increase in glycogen content in their hosts. In our study, analysis of the glycogen and 6-phosphate glucose contents verified the significant changes caused by the parasitism of *L. japonica* on *A. gossypii*. The content of glycogen in the parasitized aphids increased continuously, for providing carbohydrate nutrients for the growth and development of the parasitized aphids. 6-phosphate glucose, which is involved in Pentose phosphate pathway and glycolysis, first increased and then decreased in parasitized aphids, reflecting the changes of host metabolic targets. These changes illustrate the complexity of the relationship between *A. gossypii* and *L. japonica*, where the effects of parasitism on energy metabolism in the host appear to increase host energy reserves that, in turn, benefit development of the parasitoid [47]. The significant changes in aphid body shape and development following parasitization by *L. japonica* leads us to speculate that there is a direct relationship between the transport and metabolism of energy.

Parasitic wasps cannot synthesize lipids, and the effects of parasitization on several systems in the host lipids have been reported. Parasitism altered the lipid levels within the host and increased the metabolism of lipids and fatty acids in the hemolymph [47,48,49]. Our study produced similar results. Sterol carrier protein 2, phospholipid-transporting ATPase, acetyl-CoA acyltransferase, 3-hydroxyacyl-CoA dehydrogenase and enoyl-CoA hydratase were all induced at the protein and mRNA levels in parasitized aphids. Acyl-CoA dehydrogenases (ACADs) are a class of enzymes that catalyze the initial step in each cycle of fatty acid β-oxidation in cell mitochondria and generate glycerol and fatty acids [50]. the up-regulated proteins of acyl-CoA oxidase demonstrated the post-transcriptional regulation of gene expression. Insect parasitoids can also regulate the metabolism and development of their hosts [51]. Parasitized pea aphids displayed various degrees of castration, which have been observed in many other aphid-parasitoid systems [52]. The castration of parasitized hosts is associated with their metabolic and biochemical redirection, and helps synchronize the exponential phase of parasitoid larval growth as host nutritional supplies increase [26]. Proteins related to reproduction in *A. gossypii* significantly changed in parasitized individuals, which was consistent with previous studies. *We found that the* juvenile-hormone esterase significantly increased in parasitized aphids. FABP is released by teratocytes in A. ervi, which likely degrades aphid tissue and aids in wasp development [53,54]. Additionally, γ-glutamyl transpeptidase is a parasitoid venom that induces apoptosis in host ovarioles by generating an alteration of GSH metabolism and subsequent oxidative stress [27], however, we did not find significant changes at the protein and gene levels. These proteins or genes could be key factors in changes to the reproductive system in aphids after being parasitized by *L. japonica*. These prominent changes in genes and proteins warrant additional research.

## 4. Materials and Methods

### 4.1. Insect Materials

Live and mummified *A. gossypii* were collected from a cotton field at the Institute of Cotton Research, Chinese Academy of Agricultural Sciences (36°5′34.8″N 114°31′47.19″E). *L. japonica* were reared on *A. gossypii* at 24 ± 1 °C, 75 ± 5% relative humidity (RH) and a 14-h light:10-h dark photoperiod. A single population of parthenogenetically reproducing *A. gossypii* was reared in the laboratory on cotton leaves at 26 ± 1 °C, 65 ± 5% RH and a 14-h light:10-h dark photoperiod. To generate parasitized aphids, third-instar *A. gossypii*, from the same parent, were exposed to wasps until parasitization was observed. Both parasitized and non-parasitized aphids were reared on cotton leaves.

### 4.2. Biological Detection of A. gossypii

Freshly hatched nymphs and 1st, 2nd, 3rd, and 4th instar aphids were used during biological detection. The aphids were not classified as parasitized aphids until they became mummified. The body length and width, developmental duration, and the number of offspring of parasitized and non-parasitized aphids were recorded daily. Each individual is recorded separately, each sample had 16 replicates. SPSS 20.0 was used to evaluate the differences among copy numbers with Mann-Whitney U test (*n* = 2) and KruskaleWallis test (*n* > 2).

### 4.3. Protein Preparation and Digestion

Three-day-old parasitized and non-parasitized aphids were used for proteome analyses. After oviposition for 1 day, the wasp gradually developed into the first instar larvae, 3 days after oviposition, *L. japonica* was in the 2–3 instar larval stage.Three biological replicates of both parasitized (with larvae of *L. japonica* removed) and non-parasitized aphids were tested, with 100 individuals of each sample. Proteins were extracted from six samples by homogenization with glass beads (5 mm diameter, Sigma-Aldrich) in a freshly prepared cell lysis buffer (50 mM Tris,1 mM phenylmethylsulfonyl fluoride (PMSF), 2 mM EDTA, 2 mM dithiothreitol (DTT), pH 7.4). The suspension was sonicated for 15 min and then centrifuged at 25, 000g for 20min. We then reduced the disulfide supernatant bond of with 10 mM DTT for 1 h and blockd the cysteine with 55 mM iodoacetamide (IAM) in a dark room for 45 min, subsequently adding chilled acetone at −20°C for 2 h. The pelletin was dissolved in a 0.5 M tetrylammonium bromide (TEAB) buffer and centrifuged at 25,000× *g* for 20 min. Purified proteins (30 μg) were obtained from each sample and mixed briefly in an equivalent loading buffer at 95 °C in a heat block for 5 min, after which they were subjected to SDS-PAGE, with electrophoresis for 30 min at 80 V followed by 1.5 h at 200 V. The gel was dyed with a dyeing buffer for 2 h and destained with a destain buffer for 30 min.

We obtained 100 μg of protein for subsequent treatment from each sample solution, which was then digested with Trypsin Gold with at a protein:trypsin ratio of 20:1 at 37 for 4 h. We then added Trypsin Gold with the a protein:trypsin ratio of 20:1 againand allowed it to digest for 8 h.

### 4.4. Proteome Analysis of A. gossypii by iTRAQ

We used iTRAQ analyses from the Beijing Genomics Institute, Shenzhen, China in order to study the protein profiles of parasitized and non-parasitized aphids. After trypsin digestion, the peptides were vacuum-centrifuged until dry and treated using the ITRAQ Reagent 8-plex Kit, according to the manufacturer’s instructions. The peptides were then labeled with their respective isobaric tags and fractionated by strong cation exchange (SCX) chromatography. Chromatography was then performed using a surveyor LC system (Shimadzu LC-20AD). Data acquisition was performed with a TripleTOF 5600 System (AB SCIEX, Concord, ON, Canada) fitted with a Nanospray III source (AB SCIEX, Concord, ON, Canada) with a pulled quartz tip as the emitter (New Objectives, Woburn, MA, USA). Data were acquired using an ion spray voltage of 2.5 kV, curtain gas of 30 psi, nebulizer gas of 15 psi, and an interface heater temperature of 150 °C. The MS was operated with an RP of greater than or equal to 30,000 FWHM for TOF MS scans.

### 4.5. Protein Identification, Annotation and Quantification

During the MS/MS ions search, each query represents a complete MS/MS spectrum, and is delimited by a pair of statements. The raw MS/MS data were converted into MGF format using the corresponding tool, and the exported MGF files were searched by the local Mascot server against the database. Mascot version 2.3.02 (Matrix Science, Boston, MA, USA) was used for protein identification. Proteins were required to have at least two unique peptides for protein quantitation. Quantitative protein ratios (*p*-value < 0.05) were weighted and normalized according to the median ratio in Mascot. All proteins with a false discovery rate (FDR) less than 1% were subjected to downstream analysis. Significant proteins were determined using the parameters >1.2 or <0.8. The Blast2GO (http://www.geneontology.org) and (COG) software (http://www.ncbi.nlm.nih.gov/COG/) were used for functional classification and description. The molecular networks were annotated by KEGG database (http://www.genome.jp/kegg/pathway.html). The repeated quality of the three replicates is shown in Figure S4.

### 4.6. Proteome and Transcriptome Data Set Integration

The transcriptome data was obtained from our previous experiments, which have the ArrayExpress accession number is MTAB-5228 [55]. Proteome and transcriptome data were obtained from the same samples at the same time. Integration parameters were set as follows: Protein_FoldChange: 1; Gene_FoldChange: 2; Gene_Significant, GO_Significant, and Pathway_Significant: < 0.05. We calculated the correlation of all protein expression data of proteins related to the transcriptome level of the and Spearman’s correlation coefficient calculations. When a certain protein was detected at the transcriptome level, it is considered to be related. Protein and genetic differences associated with the cluster analysis, GO, COG, and KEGG pathway annotation were also performed with associated proteins.

### 4.7. Verification of the Contents of Glycogen and 6-Phosphate Glucose

Samples of 2–3 instar cotton aphids were obtained from cotton aphids after being parasitized for 8 h, 16 h, 1 d, 2 d, and 3 d. Samples of non-parasitized cotton aphids were obtained as controls (the larvae of wasps were removed under the microscope, and the control samples were also dissected). The contents of the glycogen and 6-phosphate glucose contents in cotton aphids parasitized for different periods of time, as well as the control, were detected using reagent kits (Glycogen Assay Kit and Glucose-6-Phosphate (G6P) Fluorometric Assay Kit, Cayman Chemical, Ann Arbor, MI, USA). SPSS 20.0 was used to evaluate the differences among copy numbers with Mann-Whitney U test (*n* = 2) and KruskaleWallis test (*n* > 2).

## 5. Conclusions

This study examined the development and physiological metabolism of *A. gossypii* parasitized by *L. japonica*. Our results demonstrated that there are strong biological changes induced by parasitism on cotton aphid, including body shape, developmental duration, and reproduction. This study provides significant insight into the proteins and genes in the aphid proteome and transcriptome that are significantly altered by the parasitoid L. japonicus. These significantly altered processes primarily relate to immunity, energy metabolism and transport, lipid metabolism and reproductive processes. We identified novel proteins and genes that respond to parasites which suggests a parasite response prior to host colonization. We verified the glycogen and 6-phosphate glucose contents and confirmed that major changes in the cotton aphids are caused by the parasitization of L. japonicus. Further characterization of these proteins will elucidate their roles as virulence factors in cotton aphid parasitism.

## Figures and Tables

**Table 1:** The morphological changes of *Aphis gossypii* parasitized by *Lysiphlebia japonica.*

Host Stage	Days after Parasitized (d)	1 Day		2 Days		3 Days		4 Days		5 Days	
		Length (mm)	Width (mm)	Length (mm)	Width (mm)	Length (mm)	Width (mm)	Length (mm)	Width (mm)	Length (mm)	Width (mm)
Larvae	CK	0.03 ± 0.02a	0.16 ± 0.01a	1.02 ± 0.12a	0.56 ± 0.06a	1.22 ± 0.12a	0.68 ± 0.11a	1.26 ± 0.08a	0.73 ± 011a	/	/
	parasitized	0.32 ± 0.01a	0.17 ± 0.01a	1.22 ± 0.14b	0.69 ± 0.09a	1.61 ± 0.13b	0.86 ± 0.11b	1.55 ± 0.12b	0.91 ± 0.14b	/	/
1st	CK	0.99 ± 0.05a	0.52 ± 0.07a	1.10 ± 0.14a	0.61 ± 0.14a	1.22 ± 0.14a	0.69 ± 0.10a	1.33 ± 0.09a	0.72 ± 0.02a	1.43 ± 0.18a	1.32 ± 0.06a
	parasitized	1.03 ± 0.04a	0.57 ± 0.04a	1.20 ± 0.05a	0.66 ± 0.02a	1.31 ± 0.12a	0.73 ± 0.12b	1.37 ± 0.08a	0.77 ± 0.03a	1.50 ± 0.01b	1.33 ± 0.05a
2nd	CK	1.04 ± 0.15a	0.58 ± 0.08a	1.20 ± 0.21a	0.67 ± 0.07a	1.34 ± 0.11a	0.75 ± 0.10a	1.41 ± 0.05a	0.75 ± 0.07a	1.43 ± 0.04a	0.81 ± 0.05a
	parasitized	1.13 ± 0.11b	0.63 ± 0.05a	1.33 ± 0.14a	0.79 ± 0.02b	1.49 ± 0.22b	0.85 ± 0.05b	1.50 ± 0.14b	0.85 ± 0.02b	1.60 ± 0.11b	0.92 ± 0.14b
3rd	CK	1.08 ± 0.08a	0.63 ± 0.08a	1.23 ± 0.11a	0.69 ± 0.02a	1.38 ± 0.18a	0.79 ± 0.01a	1.41 ± 0.25a	0.82 ± 0.01a	1.46 ± 0.11a	0.81 ± 0.01a
	parasitize	1.30 ± 0.12b	0.75 ± 0.06b	1.45 ± 0.08b	0.88 ± 0.01b	1.58 ± 0.11b	0.91 ± 0.02b	1.58 ± 0.31b	0.88 ± 0.09a	1.67 ± 0.08b	0.90 ± 0.01b
4th	CK	1.33 ± 0.14a	0.72 ± 0.01a	1.35 ± 0.09a	0.75 ± 0.01a	1.45 ± 0.11a	0.82 ± 0.08a	1.40 ± 0.11a	0.78 ± 0.04a	1.38 ± 0.04a	0.80 ± 0.02a
	parasitized	1.34 ± 0.11a	0.81 ± 0.01b	1.39 ± 0.11a	0.81 ± 0.04a	1.55 ± 0.13a	0.90 ± 0.01b	1.64 ± 0.07b	0.90 ± 0.01a	1.61 ± 0.06b	0.92 ± 0.13b
Average Increase		0.08 ± 0.01	0.07 ± 0.02	0.14 ± 0.02	0.14 ± 0.02	0.19 ± 0.03	0.11 ± 0.01	0.17 ± 0.05	0.11 ± 0.02	0.18 ± 0.01	0.09 ± 0.01

CK means Aphis gossypii of the same period that non-parasitized. The values stand for mean with standard deviation. “/” means the aphids had become mummified aphids. ‘a’ and ‘b’ indicate the significance of the same parasitic days at the same age.

**Table 2:** Developmental period of *Aphis gossypii* parasitized by *Lysiphlebia japonica.*

Host Stage		Development Period of 1st	Development Period of 2nd	Development Period of 3rd	Development Period of 4th	Development Period of Adult
Larvae	CK	27.4 ± 5.10a	28.1 ± 5.62a	29.1 ± 4.35a	/	/
	parasitied	37.2 ± 5.72b	36.0 ± 5.79b	36.6 ± 3.14b	/	/
1st	CK		17.4 ± 5.26a	30.0 ± 4.22a	28.5 ± 3.20a	/
	parasitied		27.6 ± 3.20b	39.0 ± 4.83b	36.0 ± 4.89b	/
2nd	CK			16.8 ± 3.46a	28.5 ± 3.20a	/
	parasitied			25.2 ± 3.32b	46.5 ± 4.09b	/
3rd	CK				14.0 ± 4.50a	26.9 ± 3.32a
	parasitied				19.2 ± 3.09a	39.4 ± 5.10b
4th	CK					20.9 ± 3.20a
	parasitied					36.0 ± 4.50b

CK means Aphis gossypii of the same period that non-parasitized. The values stand for mean with standard deviation. “/” means the aphids had become mummified aphids. ‘a’ and ‘b’ indicate the significance of the same parasitic days at the same age.

**Table 3:** Number of offspring of *Aphis gossypii* after parasitization by *Lysiphlebia japonica.*

	Host Stage	Larvae	1st	2nd	3rd	4th
Offspring Number	CK	34.6 ± 2.15a	34.8 ± 2.36a	31.5 ± 2.78a	33.6 ± 4.14a	29.8 ± 3.76a
	parasitized	0.67 ± 0.19d	1.68 ± 0.28cd	4.52 ± 0.67c	7.36 ± 1.02b	9.60 ± 1.12b

CK means Aphis gossypii of the same period that non-parasitized. The values stand for mean with standard deviation. ‘a’ and ‘b’ indicate the significance of the same parasitic days at the same age.

## Supplementary Materials

Supplementary materials can be found at https://www.mdpi.com/1422-0067/21/13/4610/s1.
