# Supplementary material for: Molecular Evidence that Lysiphlebia japonica Regulates the Development and Physiological Metabolism of Aphis gossypii
Source: Int J Mol Sci. 2020 Jun 29;21(13):4610. doi: 10.3390/ijms21134610 (PMC7370083; doi:10.3390/ijms21134610)
Supplement: Supplementary file 1 [file ijms-21-04610-s001.pdf]

Supplementary Information

Molecular and behavioural evidence that *Lysiphlebia japonica* regulate the development and physiological metabolism of *Aphis gossypii*

Xueke Gao, Hui Xue, Junyu Luo, LiJuan Zhang, XiangZheng Zhu, Li Wang, Shuai Zhang*, Jinjie Cui*

(Institute of Cotton Research, Chinese Academy of Agricultural Sciences / State Key Laboratory of Cotton Biology, Anyang, Henan 455000, China)

Figure S1. Identification and analysis of the proteome of parasitized aphids and non-parasitized aphids.

Figure S2. Gene ontology (GO) and clusters of orthologous groups (COG) assignments for identified proteins. Proteins were annotated in three categories.

Figure S3. GO and KEGG enrichment correlation with different expression proteins and genes.

Figure S4. Proteins quantified in three parasitized/non-parasitized aphids.

Table S1 Differentially expressed proteins and genes associated with immunization, energy metabolism and reproduction.

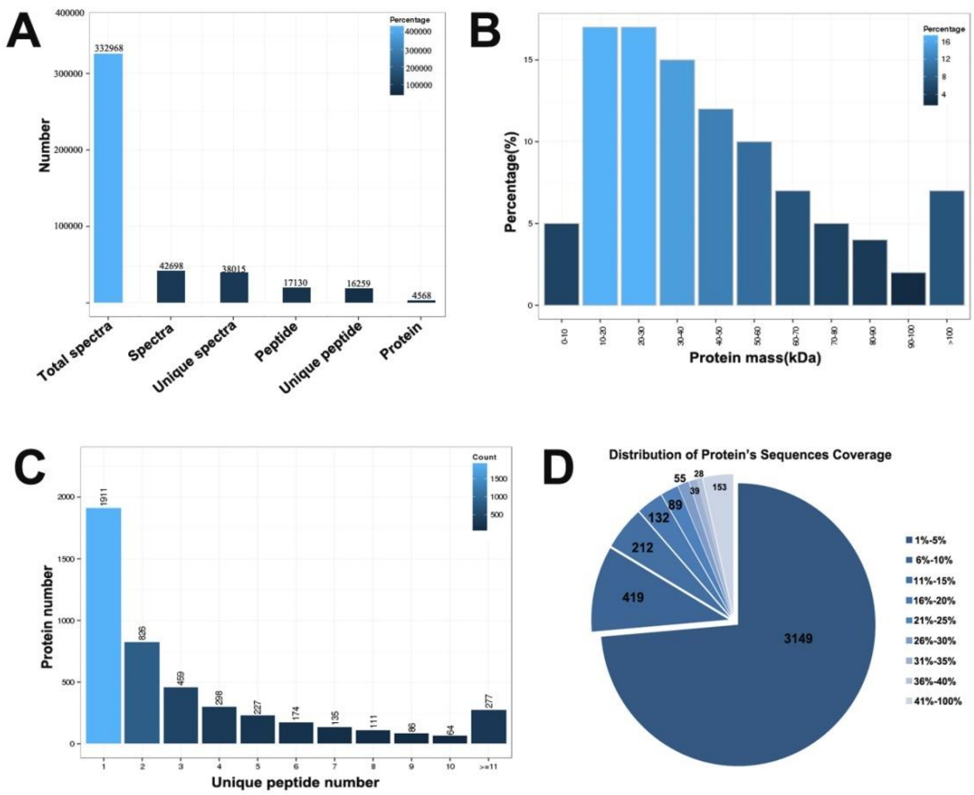

Figure S1. Identification and analysis of the proteome of parasitized aphids and non-parasitized aphids.

(A) Total spectra, spectra, unique spectra, peptides, unique peptide, and proteins identified from aphids proteomic analysis of aphids by searching protein database. (B) Identified proteins were grouped according to their protein mass. (C) Number of peptides that match proteins as indicated by MASCOT 2.3.02. (D) Pie charts classifying the identified proteins according to protein sequence coverage.

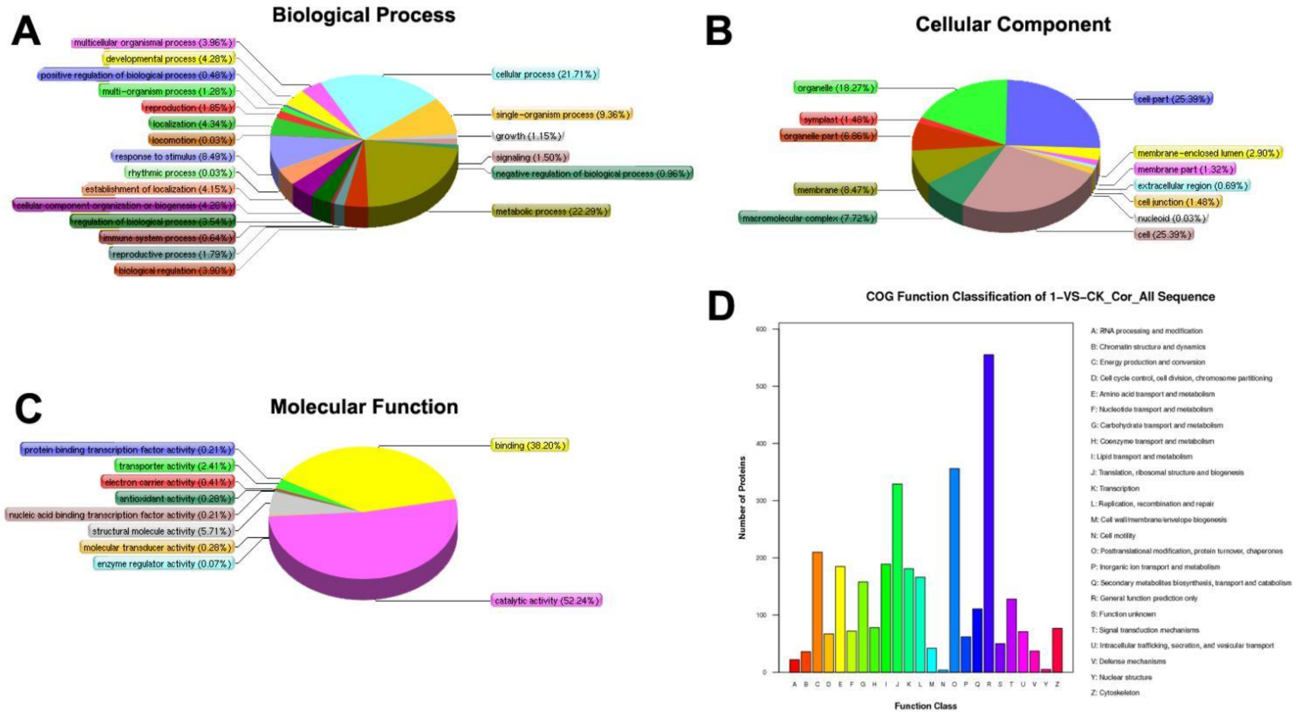

Figure S2. Gene ontology (GO) and clusters of orthologous groups (COG) assignments for identified proteins. Proteins were annotated in three categories.

(A) biological processes; (B) cellular components; (C) molecular functions; (D) Clusters of orthologous groups (COG) classification of identified proteins.

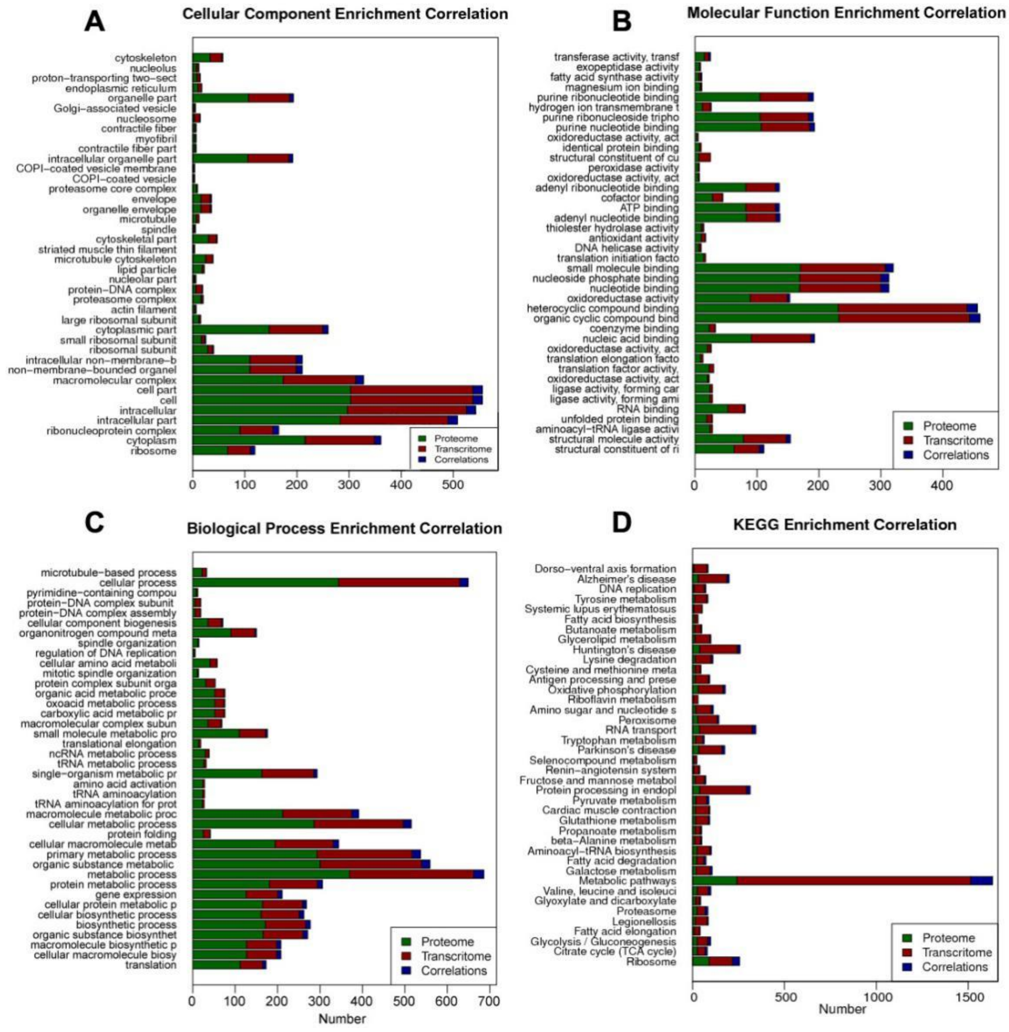

Figure S3. GO and KEGG enrichment correlation with different expression proteins and genes.

(A-C) Cellular Component, Molecular Function, Biological Process enrichment correlation of different protein and gene expressions. X-axis represents number of DEGs and DEPs. Y-axis represents GO term. (D) KEGG enrichment correlation of different protein and gene expression. X-axis represents number of DEGs and DEPs. Y-axis represents pathway name.

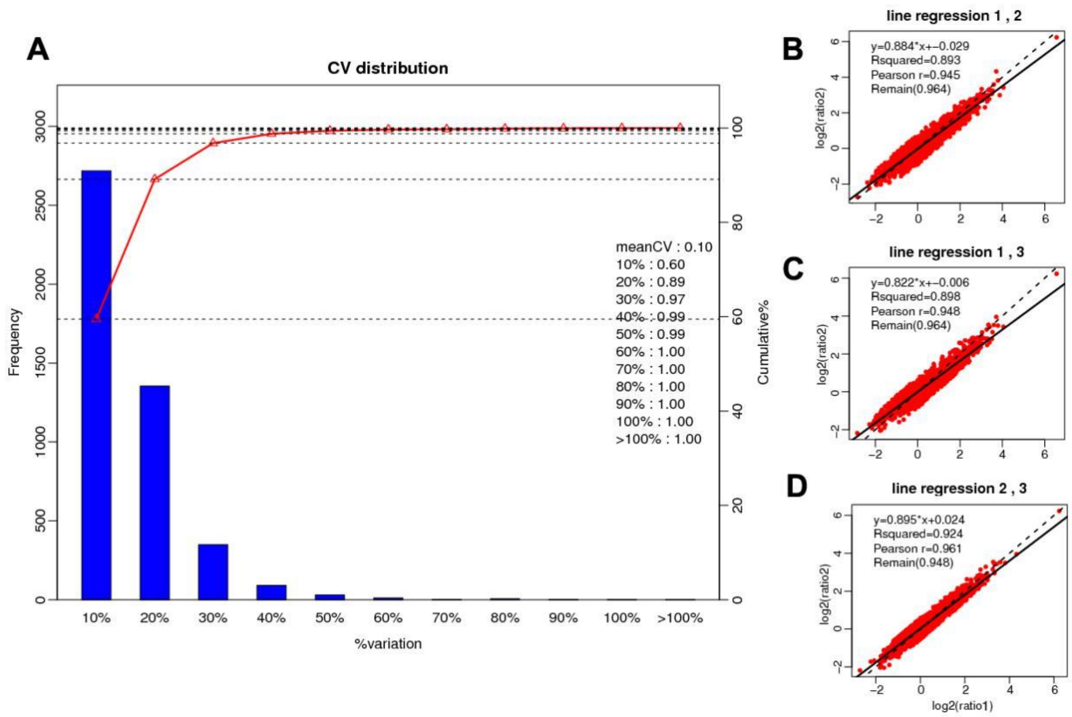

Figure S4. Proteins quantified in three parasitized/non-parasitized aphids.

(A)CV distribution in replicate. We used CV to evaluate the reproducibility. CV is defined as the ratio of the standard deviation (SD) to the mean. The lower the CV, the better the reproducibility. X-axis is the deviation between the protein ratio of the repetitive samples. Y-axis is the percentage that a protein at a certain angle comprises the amount of quantified protein.

(B)The line regression between replicates 1 and 2.

(C)The line regression between replicates 1 and 3.

(D)The line regression between replicates 2 and 3.

Table S1 Differentially expressed proteins and genes associated with immunization, energy metabolism and reproduction.

Pathway	Number	name	P_ratio(Parasitized/non-parasitized)	G_log2foldchage(Parasitized/non-parasitized)
Immunization	1	ubiquitin-conjugating enzyme E2	1.34	9.11
	2	small nuclear ribonucleoprotein D3	0.66	-1.76
	3	isocitrate dehydrogenase	2.04	11.71
	4	ubiquitin-conjugating enzyme E2 I	5.23	8.46
	5	E3 ubiquitin-protein ligase UHRF1	0.47	0.16
	6	legumain	1.80	-0.10
	7	evolutionarily conserved signaling intermediate in Toll pathways	1.76	6.10
	8	GTPase KRas	1.20	6.75
	9	filamin	2.07	4.26
	10	glycogen synthase kinase 3 beta	0.79	-0.29
	11	proliferating cell nuclear antigen	0.76	-1.67
	12	26S proteasome regulatory subunit T1	0.62	0.71
	13	glutathione S-transferaseisomerase	3.58	5.26

	14	Cu/Zn superoxide dismutase	1.67	9.91
	15	Fe-Mnsuperoxide dismutase	2.73	9.72
	16	galectin-4	1.45	4.51
	17	lectin, mannose-binding 1	1.58	10.66
	18	galectin-9	6.10	8.47
	19	lectin, mannose-binding 2	1.84	0.33
	20	serine	1.62	3.09
	21	serpin B	1.16	1.69
	22	thioredoxin domain-containing protein 10 disulfide-isomerase A1	3.48	7.48
	23	calreticulin	3.47	11.79
Reproduction	1	26S proteasome regulatory subunit T1	0.62	0.71
	2	THO complex subunit 4	0.68	0.24
	3	elongation factor 2	0.53	-0.01
	4	nucleolar protein 58	0.51	-0.68
	5	filamin	2.07	4.26

6	glycogen synthase kinase 3 beta	0.79	-0.29
7	double-strand break repair protein MRE11	0.74	-0.49
8	DNA topoisomerase II	0.59	-0.05
9	protein phosphatase 3, regulatory subunit	1.56	5.92
10	RNA-binding protein Musashi	0.78	-1.07
11	ubiquinol-cytochrome c reductase subunit 9	2.39	9.22
12	partitioning defective protein 6	0.66	-0.64
13	tropomyosin 1	2.13	6.30
14	small nuclear ribonucleoprotein D3	0.66	-1.76
15	translation initiation factor 4A	0.78	-0.14
16	small subunit ribosomal protein S28e	0.70	-0.25
17	serine/threonine-protein kinase Chk1	0.59	-1.16
18	juvenile-hormone esterase	4.10	9.91
19	fatty acid-binding protein 1	4.09	9.01
20	fatty acid-binding protein 7	4.74	10.25
21	fatty acid-binding protein 4	3.17	9.41

Amino acid and protein transport and metabolism	1	facilitated trehalose transporter	4.26	9.27
	2	glutamate synthase (NADPH/NADH)	4.10	4.79
	3	choline dehydrogenase	1.65	5.42
	4	aspartate aminotransferase, mitochondrial	2.27	9.56
	5	tyrosine 3-monooxygenase	5.28	3.60
	6	puromycin-sensitive aminopeptidase	4.45	8.44
	7	bleomycin hydrolase	2.04	7.65
	8	aminopeptidase N	3.54	6.17
	9	cystathionine gamma-lyase	2.85	8.19
	10	5-methyltetrahydropteroyltriglutamate--homocysteine methyltransferase	0.33	0.39
	11	methylenetetrahydrofolate reductase (NADPH)	0.40	0.00
	12	solute carrier family 15 (oligopeptide transporter), member 1	5.21	0.51
	13	alanine-glyoxylate transaminase / serine-glyoxylate transaminase / serine-pyruvate	0.39	0.52

		transaminase		
	14	ornithine--oxo-acid transaminase	2.41	2.33
	15	serine carboxypeptidase 1	3.34	2.47
	16	lysine-specific histone demethylase 1	0.40	5.58
	17	N4-(beta-N-acetylglucosaminyl)-L-asparaginase	2.58	7.72
	18	carboxypeptidase B	3.88	6.81
	19	isocitrate dehydrogenase (NAD+)	0.49	0.16
	20	C-terminal binding protein	2.93	7.48
	21	5-oxoprolinase (ATP-hydrolysing)	2.57	9.26
	22	translation initiation factor 3 subunit K	3.13	9.40
	23	translation initiation factor 4G	3.63	8.81
	24	ubiquitin-activating enzyme E1	3.35	7.53
Energy production and conversion	1	succinyl-CoA synthetase alpha subunit	1.57	9.57
	2	aldehyde dehydrogenase (NAD+)	2.91	9.01

3	pyruvate dehydrogenase E2 component (dihydrolipoamide acetyltransferase)	2.31	9.65
4	tumor protein p53-inducible protein 3	1.98	4.63
5	electron-transferring-flavoprotein dehydrogenase	2.64	10.07
6	pyruvate carboxylase	3.02	9.65
7	NADH dehydrogenase (ubiquinone) 1 alpha subcomplex 12	2.36	7.54
8	dihydrolipoamide dehydrogenase	2.81	9.73
9	succinyl-CoA synthetase beta subunit	3.72	9.55
10	isocitrate dehydrogenase (NAD+)	2.32	6.65
11	pyruvate dehydrogenase E1 component subunit alpha	2.36	10.61
12	citrate synthase	0.63	0.40
13	nucleosome-remodeling factor 38 kDa subunit	2.61	10.38
14	electron transfer flavoprotein beta subunit	3.15	9.93
15	F-type H+-transporting ATPase subunit alpha	2.72	11.15
16	isocitrate dehydrogenase	2.04	11.71

17	protein disulfide-isomerase A1	2.40	8.32
18	enolase-phosphatase E1	5.23	1.00
19	thioredoxin domain-containing protein 10	3.48	7.48
20	F-type H+-transporting ATPase oligomycin sensitivity conferral protein	3.21	10.24
21	malate dehydrogenase	3.21	10.13
22	NADH dehydrogenase (ubiquinone) Fe-S protein 7	2.76	8.65
23	formyltetrahydrofolate dehydrogenase	1.97	6.23
24	V-type H+-transporting ATPase subunit I	1.83	2.57
25	aconitate hydratase 1	3.64	10.17
26	alcohol dehydrogenase, propanol-preferring	2.89	9.98
27	2-oxoglutarate dehydrogenase E2 component (dihydrolipoamide succinyltransferase)	3.57	9.66
28	methylmalonate-semialdehyde dehydrogenase	0.49	0.78
29	succinate-semialdehyde dehydrogenase	2.44	5.63
30	cytochrome-b5 reductase	2.45	8.59

31	UDPglucose--hexose-1-phosphate uridylyltransferase	1.61	9.18
32	fumarate hydratase, class II	2.37	9.20
33	galactokinase	1.66	5.70
34	lysosomal alpha-mannosidase	2.09	4.53
35	facilitated trehalose transporter	4.26	9.27
36	hexokinase	2.78	9.01
37	mannose-6-phosphate isomerase	2.14	9.63
38	alpha-glucosidase	0.40	3.84
39	glyceraldehyde 3-phosphate dehydrogenase	1.82	11.61
40	GDP-L-fucose synthase	1.67	8.86
41	triosephosphate isomerase	5.85	10.49
42	ectonucleoside triphosphate diphosphohydrolase 5/6	3.89	7.93
43	phosphoacetylglucosamine mutase	2.78	7.56
44	beta-galactosidase	2.14	8.24
45	beta-mannosidase	1.98	9.78

	46	MFS transporter	4.58	5.30
Carbohydrate production and conversion	1	6-phosphofructo-2-kinase / fructose-2,6-bisphosphatase	1.66	7.23
	2	hexosaminidase	1.75	6.02
	3	MFS transporter, MCP family, solute carrier family 16	3.13	9.10
	4	UDP-N-acetylglucosamine pyrophosphorylase	3.28	7.37
	5	mannosyl-glycoprotein endo-beta-N-acetylglucosaminidase	2.52	8.24
	6	strictosidine synthase	2.20	9.44
	7	6-phosphogluconate dehydrogenase	0.48	4.30
	8	lactase-phlorizin hydrolase	1.95	9.19
	9	UTP--glucose-1-phosphate uridylyltransferase	2.07	11.29
	10	glucose-6-phosphate isomerase	3.15	7.17
	11	chitinase	3.17	8.42
	12	glucose-6-phosphate 1-dehydrogenase	2.43	8.94

	13	phosphoglucomutase / phosphopentomutase	3.37	8.42
	14	pyruvate kinase	3.02	11.73
	15	glucuronosyltransferase	5.45	8.41
	16	ribokinase	1.63	7.19
	17	1,4-alpha-glucan branching enzyme	2.75	9.28
	18	phosphoglycerate kinase	3.24	10.73
	19	NADH dehydrogenase (ubiquinone) 1 alpha subcomplex 9	0.49	0.28
Lipid transport and metabolism	1	L-gulonate 3-dehydrogenase	2.48	6.86
	2	glutaryl-CoA dehydrogenase	2.84	9.65
	3	acetyl-CoA acyltransferase	3.83	10.48
	4	glycylpeptide N-tetradecanoyltransferase	3.03	8.85
	5	acetyl-CoA synthetase	8.51	8.98
	6	4-coumarate--CoA ligase	1.91	8.88
	7	3-hydroxyisobutyrate dehydrogenase	3.74	11.27

8	3-methylcrotonyl-CoA carboxylase alpha subunit	1.72	8.98
9	3,2-trans-enoyl-CoA isomerase, mitochondrial	3.00	8.37
10	all-trans-retinol dehydrogenase (NAD+)	1.88	7.23
11	3-hydroxyisobutyryl-CoA hydrolase	3.14	9.75
12	stearoyl-CoA desaturase (delta-9 desaturase)	2.76	11.10
13	acetyl-CoA C-acetyltransferase	4.21	10.08
14	enoyl-CoA hydratase	7.28	11.39
15	3-oxoacid CoA-transferase	2.99	8.47
16	retinol dehydrogenase 8	0.46	0.28
17	Acyl-CoA dehydrogenase family member 9	2.46	7.29
18	long-chain acyl-CoA synthetase	3.17	8.94
19	retinol dehydrogenase 12	3.04	5.56
20	myo-inositol-1-phosphate synthase	0.43	0.21
21	lysophosphatidic acid acyltransferase	2.21	8.79
22	acyl-CoA oxidase	1.62	0.20
23	sterol carrier protein 2	2.94	9.33

24	3-hydroxyacyl-CoA dehydrogenase	0.34	0.61
25	NADH dehydrogenase (ubiquinone) 1 alpha	3.88	8.79
26	acyl-CoA dehydrogenase	2.79	10.44
27	phospholipid-transporting ATPase	3.62	4.86
28	1-alkyl-2-acetylglycerophosphocholine esterase	1.95	6.59
29	fatty acyl-CoA reductase	2.72	3.04
